# An anatomy of the impact of COVID‐19 on the global and intra‐Commonwealth trade in goods

**DOI:** 10.1111/roie.12637

**Published:** 2022-10-04

**Authors:** Sangeeta Khorana, Inmaculada Martínez‐Zarzoso, Salamat Ali

**Affiliations:** ^1^ Aston Business School Birmingham UK; ^2^ University of Göttingen Göttingen Germany; ^3^ University Jaume I Castellón Spain; ^4^ Marlborough House London UK

**Keywords:** COVID‐19, Commonwealth countries, gravity modelling, international trade

## Abstract

This article employs gravity modeling to examine the effect of COVID‐19 on global and intra‐Commonwealth trade. It uses bilateral monthly exports, number of COVID‐19 cases and deaths and the stringency of measures. The main novelty is the use of price indices as proxies for multilateral resistance terms, which allow us to identify, supply, and demand effects of COVID‐19 on bilateral trade. The incidence of COVID‐19 impacts Commonwealth trade flows, the effect varies with the development level. High numbers of COVID‐19 cases, including deaths, in low‐income importers reduced Commonwealth exports unlike high‐income importers that show higher exports. The incidence of COVID in an exporters' neighbouring countries impacted trade and restrictions in high‐income countries increased Commonwealth trade. Short‐term trends project a negative change in both exports and imports of Commonwealth countries.

## 1 INTRODUCTION

The COVID‐19 pandemic generated a global shock of unprecedented magnitude, with a devastating effect on international trade flows (WTO, 2020a). This followed from disruption of economic activity as a consequence of lockdowns, travel restrictions, international border and port closures, and other virus‐containment measures resulted in a macroeconomic shock. Several accounts indicate that 2020's global recession has been the worst since 1930 (Blake & Wadhwa, 2020; Hevia & Neumeyer, 2020). The pandemic affected trade flows along both supply and demand channels (Lakatos, 2020). In 2020, global trade flows collapsed on average by around 8% (WTO, 2020b); that impact, however, has varied across countries and regions, largely depending upon the level of development, trade structure, stringency of containment measures, and governments' capacity to implement policies supporting business and households.

The pandemic had a disproportionately severe economic and trade effect on 54 Commonwealth countries. It induced a deep recession in 45 Commonwealth economies, the gross domestic product (GDP) of which collapsed by US $1.45 trillion in a single year. This translated to $345 billion forgone in member countries' global exports and $60 billion in intra‐Commonwealth trade flows. Among other things, their large populations, heavy reliance on commodities exports, and similar deep recessions in major export markets, made these countries particularly susceptible to financial contagion (see Section 2). Moreover, because of pre‐existing structural vulnerabilities, COVID‐19 proved to be a particular problem for low‐income countries, including several of the commonwealth's small states. Against this backdrop, there has as yet been no detailed contextualization of the linkage between the incidence of COVID‐19 and the trade flows within the diverse group of countries that comprise the Commonwealth. The International Monetary Fund (IMF) estimated a fall in commodity prices with an adverse impact on trade and the macroeconomic situation of those countries affected (IMF, 2020). Thus, the effect of COVID‐19 on trade may be particularly acute for Commonwealth countries, which largely rely on exporting fuels, agricultural commodities, and minerals.

Given that around 70% Commonwealth countries' exports comprise goods trade, this study examines how the COVID‐19 shock has impacted bilateral merchandise trade in those countries. It employs recent advancements in empirical modeling to estimate the aggregate effect of the COVID‐19 pandemic on the global and intra‐Commonwealth trade in member countries' goods and to explore the heterogeneity (or otherwise) of that impact across various groups, including the least developed countries (LDCs) and small states. It also develops a set of policy options and recommendations aiming to revive merchandise trade flows in the Commonwealth and ensure a sustainable recovery from the economic impact of the COVID‐19 pandemic, as well as to build resilience against future shocks.

The paper uses bilateral monthly exports data from January 2019 to November 2020 to examine the short‐term effects of the COVID‐19 pandemic on global and intra‐Commonwealth trade in member countries' goods, including among these countries the LDCs and small states. We examine the exporting Commonwealth countries and their trading partners to investigate how shocks related to COVID‐19 and sector characteristics may have impacted on trade relationships. The study uses three different measures of the incidence of COVID‐19 in a country: the number of COVID‐19 infections, the number of deaths, and the stringency of measures aiming to contain the virus. This article makes important contributions. First, this is the first study to analyze the impact of COVID‐19 on the Commonwealth group of countries. A number of studies, such as Ando and Hayakawa (2022); Davidescu et al. (2022); Espitia et al. (2021); Friedt and Zhang (2021) Hayakawa and Kohei (2021); Hayakawa and Mukunoki (2020); and Masood et al. (2021) among others, use an econometric approach to examine the impact of COVID‐19 on trade. None of these, however, focuses on how the pandemic impacted *Commonwealth* trade. This article aims to fill a gap in the literature and informs discussions of the potential impact of COVID‐19 on Commonwealth merchandise trade flows, as well as offers policy recommendations to revive Commonwealth countries' economies. The second contribution is methodological, and the main novelty is the use of price indices as proxies for multilateral resistance terms, which allow us to identify, supply, and demand effects of COVID‐19 on bilateral trade. Moreover, we also account for trade frictions generated by the pandemic in countries in goods trade.

This article is structured as follows: in Section 2, we provide descriptive evidence of the impact of COVID‐19 on Commonwealth countries' economies. Section 3 presents an overview of the current literature on the economic impact of COVID‐19. Section 4 offers insight into the data and the empirical specification of the gravity model, as well as delivers our main findings. In Section 5, we draw some conclusions and make recommendations for Commonwealth countries seeking to build resilience and protect their economies against future shocks.

## 2 CORRELATIONS BETWEEN THE COVID‐19 PANDEMIC AND COMMONWEALTH COUNTRIES' TRADE FLOWS

The Commonwealth is a diverse group of 54 countries stretching from the east coast of New Zealand to the western parts of the Caribbean and South America. Six Commonwealth member countries are developed, while 48 are at various levels of economic development. This is not a formal trade bloc, but myriad drivers of international trade, such as a common language (English being a first or second language in most Commonwealth countries), a shared colonial history, some cultural common ground, and similar legal systems, underpin the trade and economic relationships within this heterogeneous association.

**FIGURE 1:**
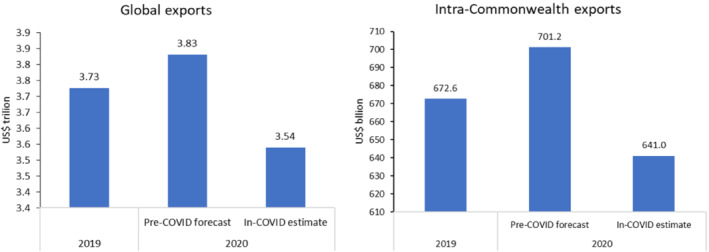
Commonwealth's global and intra‐Commonwealth exports compared, 2019 and 2020. Calculated using the IMF's pre‐pandemic forecasts (October 2019) and in‐pandemic estimates (April 2021), and trade‐to‐GDP ratios taken from the World Development Indicators. *Source*: Calculated using data from UNCTADstat [Colour figure can be viewed at wileyonlinelibrary.com]

The COVID‐19 pandemic wreaked havoc on most of the Commonwealth countries' economies. As of March 20, 2021, Commonwealth populations had succumbed to 20 million infections and witnessed 1 million deaths (Roser et al., 2020). This crisis was accompanied by a severe drop in the commonwealth's global and intra‐Commonwealth trade flows (see Figure 1). Relative to pre‐pandemic growth trends, Commonwealth economies contracted by around 10% in 2020, making this group of countries an interesting case in which to study the implications of the pandemic. While some of them have been able to contain the pandemic and resume production and trading activities, at time of writing most Commonwealth countries are still struggling to tame the pandemic.

Several factors likely contributed to the seismic trade meltdown in these economies. First, the Commonwealth is home to 32% of the world's people and its membership includes four of the 10 most populated countries (India, Pakistan, Bangladesh, and Nigeria). Aside from these demographic reasons, several underlying structural factors made these countries particularly vulnerable to the trade contagion.

**FIGURE 2:**
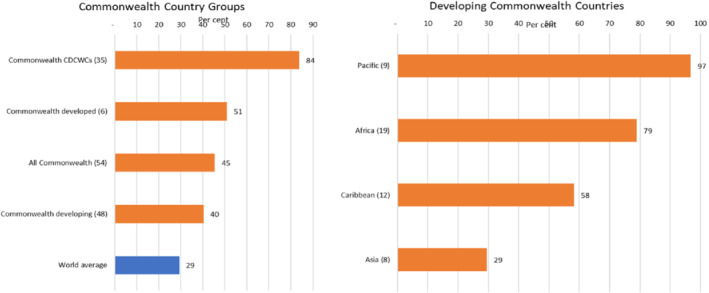
Excessive reliance of Commonwealth countries on commodities exports. *Source*: Calculated using data from UNCTADstat [Colour figure can be viewed at wileyonlinelibrary.com]

Second, two‐thirds of the commonwealth's global and intra‐Commonwealth trade is in manufactured goods and commodities. For many member countries, especially developing countries, the proportion of commodities in gross national exports ranges from 40% to more than 95%, which is extremely high compared with the world average of 29% (see Figure 2). These economies were consequently hit particularly hard when commodities prices dropped.

**FIGURE 3:**
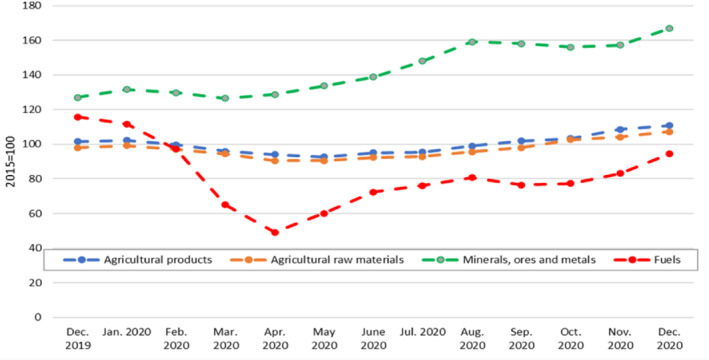
Monthly variation in commodity price indices in 2020. *Source*: Calculated using data from UNCTADstat [Colour figure can be viewed at wileyonlinelibrary.com]

Commonwealth countries' main commodity exports are food products, mineral ores, metals, and fuels. Among these, fuels are the most exported, constituting around 42% of all commodities exports. This is followed by mineral ores (36%) and agri‐food products (22%). Around 55% of these exports are destined for only five markets: China, the USA, the European Union, the United Kingdom, and Australia. The COVID‐19 pandemic negatively affected demand for commodities in these countries, leading to a collapse in commodity prices, particularly of fuels. The prices of other key commodities, such as agricultural products and mineral ores were relatively less affected. Nevertheless, a reduction in demand, along with challenges associated with production and exporting, led to an aggregate export loss of around US $125 billion for Commonwealth countries in 2020—even though the prices of most commodities had surged in the second half of 2020, offsetting some of the earlier trade losses in commodity‐dependent economies (see Figure 3).

**FIGURE 4:**
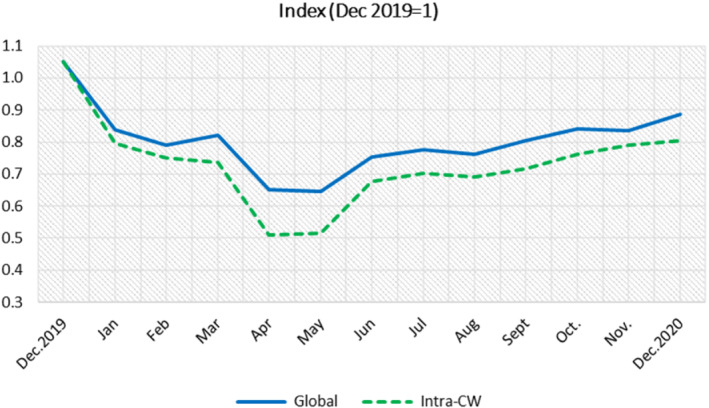
Impact of the pandemic on Commonwealth merchandise exports, December 2019 to December 2020. Exports are indexed to 1 in December 2019. *Source*: Calculated using ITC data [Colour figure can be viewed at wileyonlinelibrary.com]

The adverse effect of the pandemic on global and intra‐Commonwealth merchandise exports was first felt in early January 2020, immediately after the outbreak of the novel coronavirus in China in December 2019. The most marked effect occurred during April and May 2020, when the USA and many large export markets in Europe imposed national lockdowns. In these 2 months, Commonwealth members' exports dropped to almost half their baseline (see Figure 4). The impact was higher for intra‐Commonwealth exports compared with global exports because many of the large intra‐Commonwealth traders—including India, Singapore, South Africa, and the United Kingdom—experienced an economic contraction, affecting supply, and demand. Exports plateaued in May 2020, but they rebounded in June 2020 as firms sought to adapt to pandemic containment measures. At time of writing, merchandise exports are gradually recovering as lockdowns and other restrictions on economic activities are lifted in many countries. In December 2020, however, the commonwealth's exports were still well below their pre‐pandemic levels in December 2019.

The drop in Commonwealth countries' exports correlates strongly with incidence of the virus. Those countries with high numbers of infections (and deaths) and with strict containment measures in place are those that have experienced a large decline in trade flows.

**FIGURE 5:**
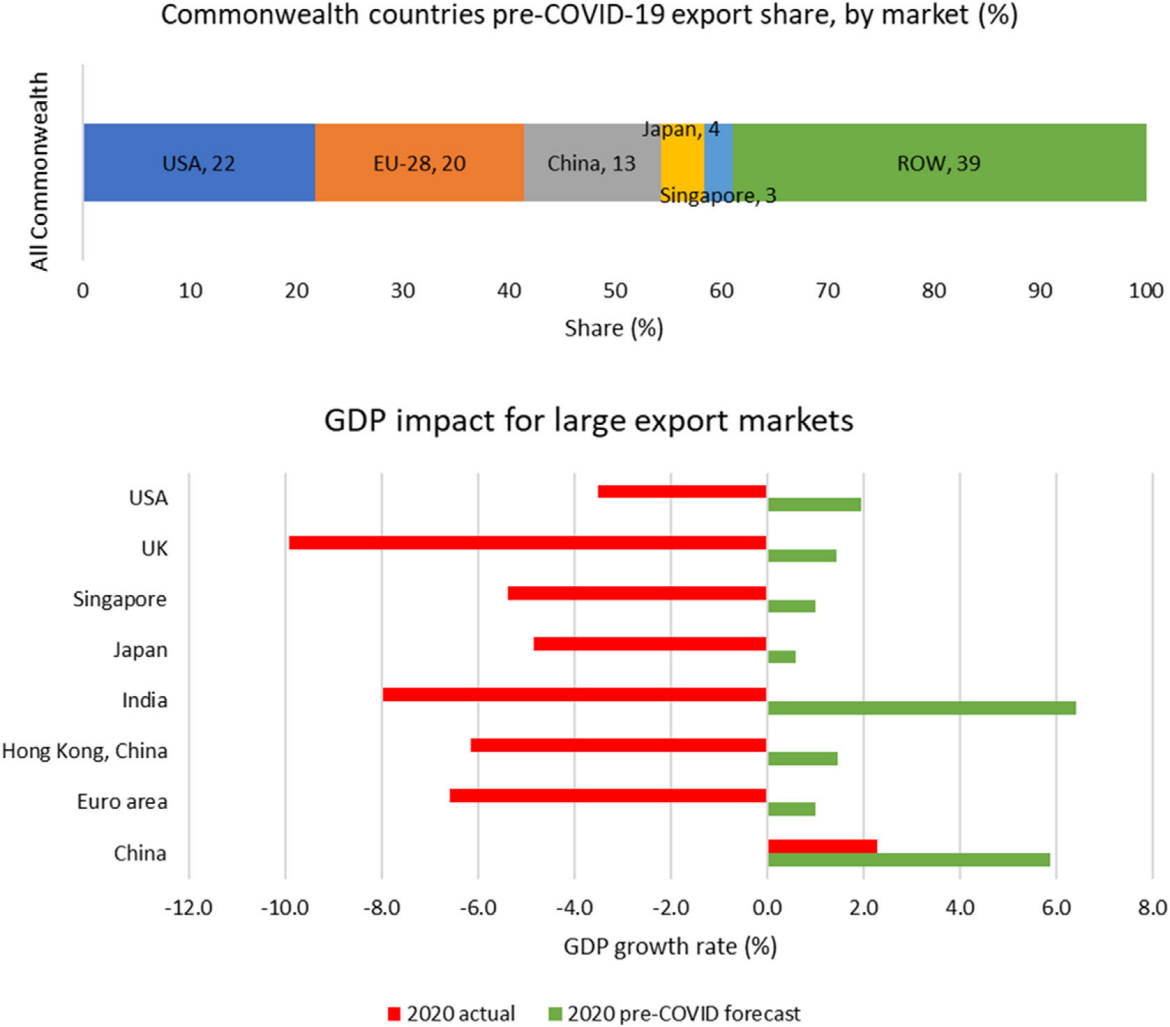
Commonwealth market share and GDP drop for large export markets (%). *Source*: Calculated using UNCTADstat and WTO‐OECD BaTIS datasets, and data from the IMF and World Bank Outlook [Colour figure can be viewed at wileyonlinelibrary.com]

Finally, the period of the pandemic saw constrained economic growth in the Commonwealth's major export markets, adversely affecting demand for goods and services (see Figure 5). Other than China, where GDP expanded by 2.3%, the major destinations for Commonwealth exports recorded significant contractions in GDP in 2020. In India and Singapore, GDP declined by more than 5%. In the USA, which absorbs 31% of developed Commonwealth members' goods and services exports and 12% of those from developing members, GDP contracted by 3.5%. The European Union, which collectively represents the second‐largest market for Commonwealth exports, contracted by 6.6%. Within the EU‐27, growth in the three top destinations for Commonwealth exports—Germany, France, and the Netherlands—fell by 4.9%, 8.2%, and 3.7%, respectively. Similarly, the GDP of the United Kingdom, a key destination for intra‐Commonwealth exports, dropped by around 9.9%. These markets collectively absorb around 75% of Commonwealth developed members' exports and around half those of developing countries. At time of writing, most of these economies are still subject to various virus‐containment measures.

## 3 LITERATURE REVIEW

The COVID‐19 pandemic resulted in an economic shock across countries that, in turn, impacted GDP and generated an economic downturn, with a negative effect on international trade. This is attributed to supply shocks in third countries that may have an impact through the competition channel as well as a demand shock in the partner country that can affect bilateral trade through the consumption channel. Studies highlighting pandemic‐related shocks to demand and supply attribute transmission of these shocks to the disruption of global value chains (GVCs) (Baldwin & Freeman, 2020; Baldwin & Tomiura, 2020). These studies suggest that the pandemic disrupted manufacturing sectors when containment efforts stifled direct supply, with impact on the international flow of intermediate inputs, and when global demand dropped as consumer spending slowed and investment was delayed.

On the demand side, Correia et al. (2020) examine the economic contagion as COVID‐19 impacted the aggregate demand channel and depressed household spending, leading to business uncertainty about future demand and an adverse effect on business investment. McKibbin and Fernando (2020) offer a supply‐side analysis of the pandemic and suggest that reduced labor supply increased the cost of production. Similarly, social distancing measures introduced to reduce the spread of the disease affected production, consumption and trade patterns, both directly and indirectly (Espitia et al., 2021). Those examining the impact of measures imposed to prevent the spread of COVID‐19 unanimously agree that the restrictions led to a sharp economic downturn. Studies examining the relationship between trade and COVID‐19 focus mainly on GVCs and whether these absorbed or transmitted COVID‐19 shocks. For example, Davidescu et al. (2022) finds that Romania, which is connected with its European partners through GVCs, is affected by changes in partner countries' relationships with the rest of the world. The authors find that Romanian exports are vulnerable to government effectiveness in relation to the other countries, as well as corruption control and cultural values. Studies by Baldwin and Tomiura (2020); Javorcik (2020a, 2020b); and Miroudot (2020) also report that GVC disruptions magnified pandemic‐induced production shock and impacted adversely on all of output, employment and trade. Others, such as Sforza and Steininger (2020); Meier and Pinto (2020); and Eppinger et al. (2020) have investigated interconnectedness and the channels through which economic shock is transmitted, finding that the economic effect of COVID‐19 was spread through supply chain linkages, with particularly severe consequences for highly integrated economies compared to those less integrated in GVCs. Eppinger et al. (2020) and Gerschel et al. (2020) also examine the global interconnectedness of international trade and GVCs, showing that slowing productivity in China's Hubei province impacted on the global economy. Friedt and Zhang (2021) employ gravity modeling to specifically examine the impact of the pandemic on Chinese exports and to explore the heterogeneity of trade effects across Chinese provinces, international trade partners and commodities. Their results show that GVC contagion reduced Chinese exports by 40%–45% during the first half of 2020—that is that Chinese exports were highly sensitive to rising rates of infection both nationally and globally. Fernandes (2020) uses difference‐in‐difference (DID) techniques to focus on trade resilience measures such as a sector's dependence on China for inputs, the labor intensity of its production and its technological proximity to other sectors.

Concerning second order effects, Bonadio et al. (2020) have examined the impact of GVC disruption on GDP. Their study differentiates between foreign and domestic shocks, and it calibrates the likely impact of lockdown measures in 64 countries by simulating what would happen if countries were to be reliant on domestic inputs. Guan et al. (2020) use the economic disaster model to assess the supply chain effects of different COVID‐19 control measures and they emphasize the indirect impacts on other countries through supply chain linkages.

Focusing on sectoral effects, Hayakawa and Mukunoki (2020) assess the correlation between the number of COVID‐19 cases and deaths and rates of bilateral exports and imports of machinery goods (finished and intermediates) between January and June 2020 for 26 reporting and 185 partner countries. Their results indicate that registered COVID‐19 cases and deaths in the exporting countries were likely to be a key factor suppressing international trade. Their findings also suggest that COVID‐19 did not impact demand for finished machinery products in the importing countries but negatively affected final machinery exports in supplier countries; as a result, supply‐side shocks were more significant in the early stages of the pandemic. A substitution effect was witnessed, results showing that a country's exports are positively associated with the pandemic burden borne by its neighbors.

Espitia et al. (2021) use the gravity model to examine how bilateral trade growth may have been impacted by supply and demand shocks during the COVID‐19 crisis among exporting, partner and third countries. This study, examining 28 exporting countries and multiple trading partners over a period from the beginning of the pandemic to June 2020, employs DID techniques and relates COVID‐19 shocks to sector characteristics. The shocks across sectors are assumed to be heterogeneous and that sector characteristics can address the decline in bilateral export growth induced by the shocks. The regression results, based on a sector‐level gravity model, show that negative trade effects induced by the shocks varied widely across sectors and that sectors within which remote working was possible contracted less than those within which it was not. Espitia et al. (2021) also find that while GVC participation increased traders' vulnerability to shocks, it also reduced their exposure to domestic shocks. Masood et al. (2021) analyze the impact of the pandemic, using a structural gravity model with the Poisson pseudo maximum likelihood estimator, for total imports as well as fruit and vegetables. The findings show a significantly negative impact of the pandemic on both import measures, which is more pronounced for the perishable goods than for aggregate imports.

Table 1 presents an overview on recent studies that analyze the impact of COVID‐19 on trade.

**TABLE 1:** Recent related studies

	Country coverage	Data for period ending 2020	Main findings
Espitia et al. (2021)	Twenty eight exporting countries (most EU members, the United States and Japan)	February–April 2020	Negative trade effects induced by COVID‐19 shocks varied widely across sectors.
Ugurlu and Jindřichovská (2022)	Visegrad countries—Czech Republic, Hungary, Poland, and Slovakia	2010–2021	COVID‐19 has a significant effect on the international trade in all the Visegrad countries
Shawn et al. (2022)	Ninety three countries	Jan 2016–December 2020	COVID‐19 reduced agricultural trade. Income level of countries matters for higher value agri‐food products, which were most impacted.
Ando and Hayakawa (2022)	Thirty five countries	January to August 2020	Investigates supply‐side effects of COVID‐19 on GVCs and reports negative supply‐side effects in three machinery industries, with the greatest effect on the transport equipment industry.
Davidescu et al. (2022)	Romania	2008–2019	Romania's export flows are vulnerable to the decrease of demand on the markets of its twelve main EU trade partners. The paper assesses the capacity of Romanian exports to regain its ascending trend displayed before the COVID‐19 pandemic by using simulation forecasting scenarios based on the shape of the economic recovery and the type of shock transmission across economies.
Webster et al. (2021)	Southern European countries	June 2020	COVID‐19 impacted all countries, cases and deaths directly affected firm sales but government containment measures, particularly closures, have more strongly affected firms
Kejžar et al. (2020)	EU‐27	May	Decline in trade due to supply and demand shocks with robust evidence of transmission of shock via backward linkages
Espitia et al. (2021)	EU‐28	June	Negative impact of COVID‐19 on trade growth with an adverse impact on demand, labor, and production
Masood et al. (2021)	OECD member states	June	Negative impact more pronounced for perishable fruits and vegetables
Hayakawa and Kohei (2021)	World	August	COVID‐19 impacted sectoral trade growth negatively with an adverse impact on GVC participation
Barbero et al. (2021)	Sixty eight developed and developing countries	October	Negative impact on trade is higher for RTA member countries especially when the exporter and importer country were at similar income levels
Khorana et al. (2021)	Commonwealth countries	November	The impact on trade varies by the level of a country's development
Liu et al. (2021)	China	December	Negative effect of COVID‐19 is most felt for medical goods and products
Arita et al. (2021)	China	December	Negative impact on aggregate agri‐trade, nonfood items and high value agri‐food most affected

*Source*: Own compilation from several studies.

Other studies have focused on the implications of COVID‐19 for trade in services, foreign direct investment (FDI), tourism, and food security. For example, Maliszewska et al. (2020) employ a standard global computable general equilibrium (CGE) model to examine the impact on trade in services by simulating the potential impact of COVID‐19 on GDP and trade. Their results show that domestic services and traded tourist services suffer the biggest negative shock. With an open‐economy model, Ozge et al. (2020) examine the macroeconomic effects of pandemic‐induced shocks on capital flows to emerging market economies. Their study shows output losses in emerging markets and attributes these losses to local currency depreciation, which has a knock‐on effect within the developed world.

International organizations have analyzed the impact of COVID‐19 on developing countries, focusing in particular on how trade in the commodity sectors has been affected (OECD, 2020b; Escaith et al., 2020; OECD, 2020a; UNECLAC, 2020). Within the context of the Commonwealth countries, Ali et al. (2020) estimated the impact of pandemic‐induced trade disruptions on exports of commodities to China, the USA, the European Union, the United Kingdom and Australia. They predicted that commodity exports to the destination markets would decline by US $98–123 billion in 2020—in percentage terms, an export loss of 19%–24% compared to pre‐pandemic benchmark estimates.

## 4 METHODOLOGY

We use the gravity model of trade to assess the impact of the COVID‐19 pandemic on bilateral trade flows. In a basic gravity model, trade between country *i* and country *j* is proportional to the size of the economies and inversely relates to the distance, a proxy for transportation costs, between them. Theoretically, the model is based on a constant elasticity substitution (CES) system. Anderson and van Wincoop (2003) used a non‐linear least squares (NLS) model that considers the endogeneity of trade costs to refine the theoretical foundations of the gravity model and provide evidence of border effects in trade. They indicated that the costs of bilateral trade between two countries are affected not only by bilateral trade costs, such as distance, landlocked, common border and languages, but also by the relative weight of trade costs in comparison to their trading partners in the rest of the world (the so‐called multilateral resistance terms). Anderson and van Wincoop (2003) derived the gravity equation in a cross‐sectional model as follows:

(1) $$x_{\mathrm{ij}}=\frac{y_{i}y_{j}}{y^{W}}{\left(\frac{t_{\mathrm{ij}}}{P_{i}P_{j}}\right)}^{1-\sigma }.$$

where *x*_*ij*_ is trade flows or exports from country *i* to country *j*. *y*_*i*_ is GDP for country *i* and *y*_*j*_ is GDP for country *j*. *t*_*ij*_ denotes trade cost between the two countries, which could be replaced by a number of trade costs proxies. *P*_*i*_ and *P*_*j*_ are the so‐called multilateral resistance terms.

As a most commonly used analytical framework, the gravity model has been applied in a large number of empirical studies to estimate the effects of trade policy changes, this is by introducing dummy variables (Egger & Nigai, 2015; Yotov et al., 2016). Among policies analyzed, a key issue is the estimation of the effects of free trade agreements (FTAs), for example Baier and Bergstrand (2009); Carrere (2006); Martínez‐Zarzoso et al. (2009), among many others.

Since the main aim of this article is to estimate the effect of the pandemic on bilateral trade, we augment the model with the number of COVID‐19 cases by country and time, the number of deaths due to the virus, and an index that is a proxy for the measures implemented during the pandemic in each country. After introducing the time dimension and the variables of interests, the first empirical specification of the model is given by:

(2) $$\begin{array}{c} X_{\mathrm{ijt}}=\exp\left(\;\beta_{1}\ln{\text{COVID}}_{\mathrm{it}}+\beta_{2}\ln{\text{COVID}}_{\mathrm{jt}}+\beta_{3}{\mathrm{lnD}}_{\mathrm{ij}}+\beta_{4}{\mathrm{FTA}}_{\mathrm{ij}}+\beta_{5}{\mathrm{COL}}_{\mathrm{ij}}+\beta_{6}{\text{BORD}}_{\mathrm{ij}}\right.\\ \left.+\beta_{7}{\text{LANG}}_{\mathrm{ij}}-\beta_{8}\ln{\mathrm{CPI}}_{\mathrm{it}}-\beta_{9}\ln\;{\mathrm{CPI}}_{\mathrm{jt}}+\theta_{i}+\pi_{j}+\gamma_{t}\right)\;\mu_{\mathrm{ijt}}, \end{array}$$

where COVID_*it*_ and COVID_*jt*_ are the number of COVID‐19 cases/deaths (or alternative stringency measures) in the respective countries at time *t*; *D*_*ij*_ denotes the great circle distance between countries *i* and *j*; FTA_*ij*_ denotes a free trade agreement dummy variable; COL_*ij*_ denotes the existence of a past or present colonial relationship between the trading countries; BORD_*ij*_ denotes adjacency of countries; LANG_*ij*_ denotes that countries have a common language; and γ_t_ indicates time‐specific effects (refer to year‐month) that are common to all trading countries.

We use the ln of the origin and destination monthly price indices, CPI_*ijt*_, which are a proxy for multilateral resistance factors that are time‐variant in the main specification. Specification (2) also includes time invariant country‐specific fixed effects, $\theta_{i}\;\text{and}\;\pi_{j}$.

The second empirical specification replaces the typical bilateral gravity variables by time‐invariant fixed effects, denoted by Φ_*ij*_, and is given by:

(3) $$X_{\mathrm{ijt}}=\exp\left(\;\gamma_{1}\ln{\text{COVID}}_{\mathrm{it}}+\gamma_{2}\ln{\text{COVID}}_{\mathrm{jt}}-\gamma_{3}\ln{\mathrm{CPI}}_{\mathrm{it}}-\gamma_{4}\ln{\mathrm{CPI}}_{\mathrm{jt}}+\Phi_{\mathrm{ij}}+\gamma_{t}\right)\;\mu_{\mathrm{ijt}}.$$

Anderson and van Wincoop pointed out that multilateral resistance factors (MRT) should be taken into account to avoid a biased estimation of the model parameters. Baier and Bergstrand (2007) used country‐pair fixed effects in addition to time‐varying trade costs to obtain unbiased estimates. In some instances, these effects should be considered as time‐variant. However, since the variables of interest are country‐time specific, adding month‐country fixed effects will impede us to directly estimate the coefficients of the COVID variables. The identification strategy followed by Barbero et al. (2021) was to interact these target variables with the FTA membership. In this way, the authors identify the differential effect for countries being in an FTA with respect to non‐FTA countries.^1^ In this article we use consumer price indices that are available at a monthly base as proxies for multilateral resistance.

Since it is not only in the source and destination country where the pandemic matters, but also countries where goods have to transit. COVID‐19 leads to trade frictions through disruption of transport, to account for this we proceed as follows: We analyze the impact of COVID‐19 on neighboring exporting and importing countries in terms of bilateral exports of a given trading pair. The number of cases/deaths in the neighboring countries, calculated using a distance‐weighted sum of COVID‐19 burden, is given by:

(4) $${\text{NeighCOVID}}_{\mathrm{it}\left(\mathrm{jt}\right)}=\sum_{j\neq i}\left(\frac{{\text{COVID}}_{\mathrm{jt}\left(\mathrm{it}\right)}}{{\text{Distance}}_{\mathrm{ij}}}\right),$$

where COVID_*it*(*jt*)_ represents the number of cases and number of deaths in country *i(j)*.

The empirical specification of the model, given by Equation (2) above, is augmented with the corresponding variables:

(5) $$\begin{array}{c} X_{\mathrm{ijt}}=\exp\left(\delta_{1}\ln{\text{COVID}}_{\mathrm{it}}+\delta_{2}\ln{\text{COVID}}_{\mathrm{jt}}+\delta_{3}\ln{\text{NeighCOVID}}_{\mathrm{it}}\right)\\ \left.+\delta_{4}\ln\text{Neigh}{\text{COVID}}_{\mathrm{jt}}+\delta_{5}\ln\;{\mathrm{CPI}}_{\mathrm{ijt}}+\delta_{6}\ln\;{\mathrm{CPI}}_{\mathrm{ijt}}+\phi_{\mathrm{ij}}+\gamma_{t}\right)\;\mu_{\mathrm{ijt}}. \end{array}$$

Note: please see the definition of other variables is under Equation (2).

According to recent developments, the models are estimated applying a Poisson pseudo maximum likelihood (PPML) approach for the multiplicative model to retain the zero trade flows (Head & Mayer, 2014; Yotov et al., 2016).

It is important to notice that the use of monthly data presents challenges. For instance, although the micro‐founded gravity model relays general equilibrium restrictions, namely, market clearing, which does not have to hold on a monthly basis and has implications for the variables included as regressors in the gravity model.^2^ Further, while GDP is not measured as a monthly frequency this is not the case with prices, which are on a monthly basis.

### 4.1 Data sources and variables

The main source for bilateral trade flows is monthly data from the UN Comtrade database for January 2019 to November 2020. See Table A1 for a list of Commonwealth countries.

Health authorities worldwide have collected primary data on COVID‐19 on a daily basis. The data on the number of COVID‐19 cases, number of deaths and the stringency index is retrieved from Roser et al. (2020). Data on GDP in nominal values and population in number of inhabitants is obtained from the World Bank Development Indicators data series. The data on geographical and cultural proximity, such as distance, shared border and common language, is from the Center d'Etudes Prospectives et d'Informations Internationales (CEPII) database. The monthly consumer price indices (year base 2015) are from FAOSTAT. Table 2 presents some summary statistics.

**TABLE 2:** Summary statistics

Variable	Obs	Mean	Std dev	Min	Max
Trade value (US$)	213,380	1.025e + 08	7.982e + 08	0	4.713e + 10
Partner GDP 2019, constant 2010	206,439	6.568e + 11	2.070e + 12	12,581	1.830e + 13
Reporter GDP 2019, constant 2010	213,349	9.698e + 11	2.602e + 12	18,008	1.830e + 13
Partner population 2019	207,605	55,230,309	1.784e + 08	11,646	1.398e + 09
Reporter population 2019	206,805	59,541,509	1.838e + 08	18,008	1.366e + 09
Partner new monthly cases	213,380	10,331.793	93,175.493	0	4,496,410
Partner monthly deaths	213,380	291.381	2290.678	0	60,750
Partner monthly average stringency	213,380	16.473	29.057	0	100
Reporter total monthly cases	213,380	19,659.207	152,796.27	0	2,621,418
Reporter total monthly deaths	213,380	523.002	3368.124	0	60,750
Reporter monthly average stringency	213,380	15.647	27.556	0	100
Contiguity dummy	213,380	0.024	0.154	0	1
Common language dummy	213,380	0.134	0.341	0	1
Former colony dummy	213,380	0.022	0.148	0	1
Distance between countries	213,380	6983.341	4354.057	19.127	19,812.043
Monthly ln consumer price index partner	175,415	4.781	0.263	4.573	8.258
Monthly ln consumer price index reporter	195,083	4.742	0.195	4.583	6.947

### 4.2 Main results

To examine how COVID‐19 impacted international trade we analyzed monthly trade data for 186 countries from January 2019 to November 2020. Table 3 presents the main results for the whole sample.

**TABLE 3:** Main results: gravity model estimations with PPML for the whole sample

Dep. variable:	(1)	(2)	(3)	(4)	(5)	(6)
Export value	COVID cases	COVID deaths	Stringency index	COVID cases	COVID deaths	Stringency index
Explanatory variables:						
lncovid measure i, t‐1	−0.020***	−0.016**	−0.002	−0.012***	−0.007	−0.005
	(0.006)	(0.006)	(0.007)	(0.004)	(0.005)	(0.006)
lncovid measure j, t‐1	0.001	0.001	−0.005	0.001	0.001	−0.006
	(0.005)	(0.005)	(0.004)	(0.005)	(0.005)	(0.004)
lndij	−0.669***	−0.669***	−0.669***			
	(0.040)	(0.040)	(0.040)			
Regional trade	0.459***	0.459***	0.459***			
agreement dummy	(0.084)	(0.084)	(0.084)			
Former colony dummy	0.313***	0.312***	0.312***			
	(0.119)	(0.119)	(0.120)			
Contiguity dummy	0.392***	0.392***	0.391***			
	(0.096)	(0.096)	(0.096)			
Common language	0.024	0.023	0.024			
dummy	(0.103)	(0.103)	(0.103)			
lnCPIi	−0.378*	−0.329	−0.390*	−0.190	−0.169	−0.197
	(0.205)	(0.201)	(0.207)	(0.135)	(0.134)	(0.136)
lnCPIj	−0.557**	−0.557**	−0.551**	−0.520**	−0.525**	−0.527**
	(0.230)	(0.233)	(0.229)	(0.230)	(0.235)	(0.232)
Observations	152,601	152,601	152,601	151,768	151,768	151,768
Year‐Month FE	Yes	Yes	Yes	Yes	Yes	Yes
i, j FE	Yes	Yes	Yes			
Pseudo R^2^	0.917	0.917	0.917	0.991	0.991	0.991
ij FE				Yes	Yes	Yes

*Note*: Robust standard errors in parentheses. ***p < .01; **p < .05; *p < .1.

The gravity model has been estimated using PPML: first, with price indices as proxies for the multilateral resistance terms (MRT) and time‐invariant gravity variables with Equation (2); second, with bilateral fixed effects (FE) instead of time‐invariant bilateral variables with Equation (3). The first three columns of Table 3 report the results of estimating Equation (2) using PPML and show the estimated coefficient of COVID‐19‐related and gravity variables. All the COVID‐19‐related variables are taken with one lag ‐ that is the previous month—to account for lagged effects.

The coefficients indicate that a 10% increase in the number of COVID‐19 cases in the exporter country decreases exports by 0.2% (column 1). A slightly lower effect is found for the number of deaths in the exporting country (0.016, column 2). However, the effect on exports is not significant when the number of cases/deaths increase in the importer country. The results for the stringency index cannot be confirmed, being the corresponding coefficient nonsignificant at conventional levels (column 3). The effects decrease in magnitude and loss significance—with only the number of COVID‐19 cases in the exporter being statistically significant but showing a smaller effect—when the gravity variables, namely, distance, FTA, common language, and common border are replaced by bilateral fixed effects (columns 4 to 6). All gravity variables present the expected sign and, with the exception of language, all are statistically significant at conventional levels.

We also estimate a log‐linearized version of the model with the ordinary least squares (OLS) that includes income and population effects. The results are presented in the Appendix (Table A2). The GDP, population and gravity variables are included in the traditional gravity model (columns 1–3) and a similar specification with bilateral FE for comparison in columns (4)–(6). The results also indicate a negative effect of COVID incidence in the exporting countries. For a comparison of results obtained from OLS and PPML estimations, comparable models are estimated in Table A3. The PPML estimated coefficients for the target variables present the same sign as the OLS but are significantly higher in magnitude (compare columns 1 and 2 with columns 3 and 4).

As stated in the methodological section, it is important to account for trade frictions generated by the pandemic in countries through which goods trade. The estimates in Table 4 show that an increase in the number of COVID‐19 cases in the countries neighboring the exporter has a negative and significant effect on exports that is almost twice the estimated effect of COVID incidence in the exporting country in column (1) (compare −0.53, with −0.027) and this is the same for the number of deaths. The effects stay similar when controlling for pair FE in columns (4) and (5) for cases and deaths, respectively. Lockdowns and other containment measures in the countries that are geographically close to the exporter also exert an additional impact on exports that is significant at the 10% and 5% levels in columns (3) and (6), respectively. This indicates the importance of supply side shocks affecting exports. However, the effect of COVID incidence, deaths, and stringency measures in countries neighboring the importer is only significant for the first two in columns (1) and (2), but loses significance when we control for all pair‐time‐invariant factors in columns (4) and (5). This suggests that the demand mechanism works in this case through other unobserved bilateral factors that affect trade cost.

**TABLE 4:** Gravity model adding COVID‐19 incidence in neighboring countries

Dependent variable:	(1)	(2)	(3)	(4)	(5)	(6)
Export value	COVID cases	COVID deaths	Stringency index	COVID cases	COVID deaths	Stringency index
Explanatory variables:						
lncovid measure i, t‐1	−0.027***	−0.025***	−0.005	−0.015***	−0.012**	−0.004
	(0.007)	(0.007)	(0.007)	(0.005)	(0.005)	(0.006)
lncovid measure j, t‐1	−0.006	−0.008*	−0.012**	−0.005	−0.006	−0.012**
	(0.005)	(0.005)	(0.005)	(0.005)	(0.004)	(0.005)
lnneig cov i, t‐1	−0.053***	−0.060***	−0.093*	−0.047***	−0.055***	−0.108**
	(0.015)	(0.017)	(0.053)	(0.014)	(0.017)	(0.051)
lnneig cov j, t‐1	−0.098**	−0.117***	−0.060	−0.046	−0.050*	0.046
	(0.038)	(0.037)	(0.073)	(0.032)	(0.029)	(0.048)
Lndij	−0.670***	−0.670***	−0.669***			
	(0.040)	(0.040)	(0.040)			
Regional trade agreement dummy	0.459***	0.459***	0.459***			
	(0.084)	(0.084)	(0.084)			
Former colony dummy	0.313***	0.313***	0.312***			
	(0.119)	(0.119)	(0.120)			
Contiguity dummy	0.390***	0.391***	0.391***			
	(0.096)	(0.096)	(0.096)			
Common language dummy	0.023	0.023	0.024			
	(0.103)	(0.103)	(0.103)			
lnCPIi	−0.336*	−0.272	−0.397*	−0.166	−0.141	−0.190
	(0.204)	(0.200)	(0.206)	(0.134)	(0.133)	(0.134)
lnCPIj	−0.521**	−0.501**	−0.547**	−0.474**	−0.460**	−0.515**
	(0.220)	(0.216)	(0.229)	(0.215)	(0.215)	(0.230)
Observations	152,601	152,601	152,601	151,768	151,768	151,768
Year‐Month FE	Yes	Yes	Yes	Yes	Yes	Yes
i and j FE	Yes	Yes	Yes			
Pseudo R^2^	0.917	0.917	0.917	0.991	0.991	0.991
ij FE				Yes	Yes	Yes

*Note*: Robust standard errors in parentheses. ***p < .01; **p < .05; *p < .1.

The magnitude of the coefficients of price indices is lower in Table 4 in comparison to Table 3. A possible explanation is that part of the COVID effect on exports could have been transmitted through changes in consumer prices, when not controlling for the effects in neighboring countries.

As a next step we investigate the existence of heterogeneity in the effects of the target variables due to income differences between trading countries. Table 5 distinguishes between high‐income countries^3^ and the other countries. Given that many developed countries initiated support mechanisms to cope with the effects of the pandemic, we expect to find a heterogeneous effect on exports depending on whether the cases increase in developed or developing countries. On the import side, we expect the effects of COVID‐19 to be stronger for developing countries where governments were not always able to financially support the populations as was the case in most developed economies.

**TABLE 5:** Gravity model estimations: heterogeneous effects by income level

Dependent variable:	(1)	(2)	(3)
Export value	COVID cases	COVID deaths	Stringency index
Explanatory variables:			
lncovid measure i, t‐1	−0.012***	−0.009*	−0.005
	(0.004)	(0.005)	(0.007)
lncovid measure j, t‐1	−0.011**	−0.016***	−0.030***
	(0.005)	(0.004)	(0.005)
HI*lncovid measure i, t‐1	0.002	0.004	0.002
	(0.002)	(0.003)	(0.006)
HI*lncovid measure j, t‐1	0.014***	0.020***	0.038***
	(0.002)	(0.003)	(0.005)
lnCPIi	−0.123	−0.095	−0.155
	(0.112)	(0.115)	(0.123)
lnCPIj	−0.092	−0.132	−0.097
	(0.126)	(0.134)	(0.126)
Observations	145,331	145,331	145,331
Year‐Month FE	Yes	Yes	Yes
i, j FE	Yes	Yes	Yes
Pseudo R^2^	0.991	0.991	0.991

*Note*: Robust standard errors in parentheses. ***p < .01; **p < .05; *p < .1.

Abbreviation: HI, high‐income countries.

The results of estimating model (3) show that the exports decrease when the number of COVID‐19 cases in the importing country increase, and this is especially the case for low‐income importing countries.^4^ On the demand side, low‐income countries spend a much larger share of household budgets on food, which explains why the purchases are more sensitive to income changes that may be caused by COVID‐19. On the supply side, low‐income countries may also be more vulnerable to supply chain disruptions. However, when the importer is a high‐income country (HI) the effect is positive and significant, indicating that countries with higher numbers of COVID‐19 cases import more from the rest of the world, due to increased demand from abroad during lockdown. When we consider the incidence of COVID on the exporters' side, the results are not statistically different for high‐income countries and other countries and are now statistically significant, whereas those were not in Table 3. A comparable pattern is observed when the target variable is the number of COVID‐19 deaths (column 2) or the stringency index (column 3), with the only difference that the effects for COVID deaths/stringency in the exporter is not statistically significant.

In Table 6, the gravity model is estimated for the Commonwealth exporting and importing countries separately; the results are on the left‐ and right‐hand sides of the table, respectively.

**TABLE 6:** Model estimated for Commonwealth exporters and importers

Dependent variable:	(1)	(2)	(3)	(4)	(5)	(6)
Export value	CW exports			CW imports		
Explanatory variables:	COVID cases	COVID deaths	Stringency index	COVID cases	COVID deaths	Stringency index
lncovid measure i, t‐1	−0.002	−0.004	−0.017	−0.004	−0.001	−0.002
	(0.005)	(0.006)	(0.015)	(0.006)	(0.007)	(0.013)
lncovid measure j, t‐1	−0.016**	−0.019**	−0.025**	−0.009**	−0.017***	−0.023*
	(0.007)	(0.007)	(0.012)	(0.004)	(0.005)	(0.013)
HI*lncovid measure i, t‐1	−0.008	−0.017**	−0.017	−0.004	−0.004	−0.013
	(0.006)	(0.007)	(0.015)	(0.005)	(0.007)	(0.010)
HI*lncovid measure j, t‐1	0.017***	0.024***	0.046***	0.024***	0.031***	0.052***
	(0.003)	(0.005)	(0.010)	(0.005)	(0.007)	(0.009)
lnCPIi	−1.732***	−1.590***	−1.732***	−0.156	−0.129	−0.170
	(0.479)	(0.501)	(0.469)	(0.232)	(0.229)	(0.235)
lnCPIj	−0.025	−0.041	−0.020	−0.231	−0.540	−0.404
	(0.140)	(0.146)	(0.135)	(0.462)	(0.445)	(0.460)
Observations	35,103	35,103	35,103	31,638	31,638	31,638
Year‐Month FE	Yes	Yes	Yes	Yes	Yes	Yes
i, j FE	Yes	Yes	Yes	Yes	Yes	Yes
Pseudo R2	0.990	0.990	0.990	0.988	0.988	0.988

*Note*: Robust standard errors in parentheses. ***p < .01; **p < .05; *p < .1.

Abbreviation: HI, high‐income countries.

As in Table 4, we add the interactions for COVID‐19 variables with a dummy variable for high income Commonwealth countries to acknowledge that the effects can be heterogeneous. The estimates indicate that a high incidence of COVID‐19 in the low‐income importing countries (ln covid measure j, t‐1) reduces Commonwealth exports, whereas a high incidence in the high‐income importing countries (HI) increases Commonwealth exports (see columns 1 to 3). The number of COVID‐19 cases in the exporting countries, however, plays a minor role. When the focus is on Commonwealth imports (columns 4 to 6) it is important to note that only the incidence of COVID‐19 in the importing countries plays a role and the coefficients are slightly higher in magnitude than for exports in the case of HI countries.

Table 7 presents similar estimates for intra‐Commonwealth trade.

**TABLE 7:** Results for intra‐Commonwealth trade by developed and developing countries

Dependent variable:	(1)	(2)	(3)	(4)	(5)	(6)
Export value	COVID cases	COVID deaths	Stringency index	COVID cases	COVID deaths	Stringency index
Explanatory variables:						
lncovid measure i, t‐1	0.010	0.019	−0.000	−0.005	0.002	−0.033
	(0.013)	(0.016)	(0.032)	(0.010)	(0.011)	(0.031)
lncovid measure j, t‐1	−0.012	−0.017*	−0.032	−0.012	−0.017**	−0.022
	(0.009)	(0.010)	(0.030)	(0.007)	(0.008)	(0.027)
HI*lncovid measure i, t‐1	−0.010	−0.005	−0.028	0.003	0.015	0.000
	(0.013)	(0.023)	(0.027)	(0.006)	(0.010)	(0.014)
HI*lncovid measure j, t‐1	0.033***	0.048***	0.062***	0.032***	0.051***	0.054***
	(0.008)	(0.014)	(0.017)	(0.007)	(0.011)	(0.014)
lndij	−0.588***	−0.586***	−0.590***			
	(0.100)	(0.100)	(0.100)			
RTA dummy	0.946***	0.948***	0.945***			
	(0.174)	(0.174)	(0.174)			
Former colony dummy	1.454***	1.454***	1.454***			
	(0.481)	(0.481)	(0.482)			
Contiguity dummy	−0.932**	−0.929**	−0.934**			
	(0.456)	(0.457)	(0.456)			
Common language dummy	2.415***	2.403***	2.420***			
	(0.354)	(0.354)	(0.353)			
lnCPIi	−0.773	−0.784	−0.777	−1.743***	−1.854***	−1.843***
	(1.086)	(0.984)	(1.173)	(0.641)	(0.590)	(0.692)
lnCPIj	−1.649*	−1.904**	−1.993**	−1.602*	−1.799*	−2.034**
	(0.923)	(0.904)	(0.944)	(0.947)	(0.923)	(0.957)
Constant	33.334***	34.586***	35.016***	35.559***	37.018***	38.108***
	(6.519)	(5.968)	(7.065)	(5.408)	(5.018)	(5.665)
Observations	8332	8332	8332	8275	8275	8275
Year‐Month FE	Yes	Yes	Yes	Yes	Yes	Yes
i, j FE	Yes	Yes	Yes	Yes		
Pseudo R2	0.902	0.902	0.902	0.980	0.980	0.980
ij FE				Yes	Yes	Yes

*Note*: Robust standard errors in parentheses. ***p < .01; **p < .05; *p < .1.

Abbreviation: HI, high‐income countries.

The first part of the table presents the results of gravity and COVID‐19 variables, whereas the second replaces the gravity variables with time‐invariant bilateral FE as in Table 3 for the whole sample. In general, the effect of COVID‐19 on intra‐Commonwealth trade is significant for low‐income countries when considering the incidence in terms of the number of deaths in the importing countries (compare 0.016 in Table 4, column 2, with 0.0017 in Table 6, column 5 for low‐income countries). The stringency index also presents a different effect for Commonwealth trade, that is in high‐income Commonwealth countries a higher level of stringency measures increases trade (0.054, column 6 in Table 6, versus 0.038 in column 3, Table 4).

### 4.3 Robustness checks and simulations

For robustness, first we consider the estimation of an alternative specification in which MRT is specified using exporter‐time and importer‐time dummy variables. In order to identify the coefficients of our target variables in the model, we interact the centered‐distance variable with the COVID factors. In this way we will be able to obtain estimates indicating whether COVID incidence in countries that are above the average distance from the given exporter have a relative lower effect on exports. Moreover, we will also be able to compare those estimates with the ones obtained when CPI are used as proxies for MRT. The main results, shown in Table 8, indicate that the coefficients obtained for the interaction variables barely change when using MRT dummy variables (column 2) in comparison with when using the consumer price indices. This similarity indicates that CPIs are good proxies for MRT.

**TABLE 8:** Comparing gravity model with CPI and multidimensional FE

Dependent variable:	(1)	(2)
Export value	With CPI	With multiD FE
Explanatory variables:		
lncovid cases i, t‐1	−0.015***	
	(0.004)	
lncovid cases j, t‐1	0.000	
	(0.005)	
Lnd*covidcai	0.005**	0.004**
	(0.002)	(0.002)
Lnd*lncovidcaj	−0.004**	−0.003*
	(0.002)	(0.002)
lnCPIi	−0.187	
	(0.133)	
lnCPIj	−0.465**	
	(0.216)	
Observations	174,359	195,083
Year‐Month FE	yes	
it, jt FE		Yes
ij FE	Yes	Yes
Pseudo R2	0.991	0.996

*Note*: Robust standard errors in parentheses. ***p < .01, **p < .05, *p < .1.

Concerning the interpretation of the estimates, whereas the coefficient is positive from the supply side (Lnd*lncovidca), meaning that the effect of an increase in COVID cases in the exporting country is smaller (less negative) for countries further away from the exporter, the effect is negative from the demand side, indicating that for distances above the average, the effect of an increase in COVID cases in the importer country is reinforced for countries nearby.

Second, we consider a control function approach to deal with the potential errors in variables issue. In the first step we estimate a model with the COVID factors (incidence/deaths) as dependent variables and containment factors as explanatory variables and save the residuals, which are then added to the PPML gravity model as additional regressors in a second step. The second step is estimated with bootstrapped standard errors and 10 replications. The main results indicate that the added terms from step one estimations are not statistically significant and hence, the correction does not change the main results for the target variables.^5^

## 5 CONCLUSIONS AND POLICY IMPLICATIONS

This article uses gravity modeling to examine the link between bilateral trade flows for Commonwealth exports and the impact of COVID‐19 on the global and intra‐Commonwealth trade in goods. Analysis of data spanning January 2019 to November 2020 suggests that COVID‐19 had an adverse impact on trade and that exports decreased as the number of COVID‐19 cases rose in an importing country—that is that high COVID‐19 incidence in low‐income importing countries reduces Commonwealth exports, whereas high COVID‐19 incidence in high‐income importing countries increases Commonwealth exports. The incidence of COVID‐19 in the exporting country, however, does not impact on trade. For Commonwealth imports, the incidence of COVID‐19 in importing countries also has an effect. In high‐income importing Commonwealth countries, an assessment of demand effects suggests that more stringent measures aiming to contain the virus are associated with increased trade.

The pandemic is ongoing at time of writing, and there is uncertainty about its likely duration and severity across countries and regions. The pandemic has also revealed the vulnerability of Commonwealth countries linked in GVCs, with supply and demand shocks having had a ripple effect. In this context, Friedt and Zhang (2021) suggest that governments' policy response must aim at increasing the resilience of GVCs—that policy‐makers must devise measures that protect economies against supply chain shocks and build their resilience. An important point to note is that, to design effective and co‐operative policies as part of any recovery initiative within the Commonwealth, co‐operation is required at the regional and global levels.

To address the vulnerability of countries linked in GVCs, commodity‐dependent Commonwealth countries should consider a set of policies and investments targeting inclusive structural transformation and aiming to diversify the economy. At the same time, commodity‐dependent countries should consider adopting policy frameworks and measures that support a sustainable recovery post‐COVID‐19 and which build resilience against future shocks.

Short‐term measures to overcome the challenges of COVID‐19 can be linked to economic growth by investing in productivity and policies aiming to enhance the resilience of Commonwealth countries. Appropriate planning is required to minimize the impact on sectors linked in GVCs. A roadmap will help countries to achieve their short‐, medium‐ and long‐term goals and to revitalize their economies by taking into account the specific conditions and needs of those sectors adversely affected. In the short term, governments should focus on the immediate health crisis, on ensuring food and nutritional security, on job creation and on supporting the economy to ensure that there is no long‐term scarring from the pandemic.

In the medium term, Commonwealth countries' focus should be on boosting bounce‐back activities that will transform the recovering economy by promoting the long‐term sustainable growth of international trade. For example, regional co‐operation might be one way of achieving an inclusive structural transformation. An important driver for co‐operation in Africa might be the African continental free trade area (ACFTA), which can add value and support diversification, especially by means of participation in regional value chains.

Finally, in light of their growing participation in world trade, Commonwealth countries might find in the current situation a unique opportunity to use new technologies to support policies targeting recovery. The use of new technologies, such as additive manufacturing will prompt a restructuring of GVCs and may mitigate risks by means of a combination of diversification strategies. It is also possible, however, that automation will fuel production reshuffling that shifts nations' incentives and yields a redistribution of manufacturing around the globe.

To conclude, while Commonwealth countries will focus in the short term on remedying the adverse impacts of the pandemic and restoring jobs and employment, their long‐term focus may be on improving productivity and boosting their resilience by investing in a balanced portfolio of physical, human, social and natural capitals. For example countries may choose to invest in health, education, skills development, innovation, technological upgrading, and green infrastructure and natural capital, thereby increasing the productive capacity of the population and providing sustainable returns for future generations. In this way, Commonwealth countries may build capacity to deal with future challenges and mitigate the impact of future crises, including pandemics, and other socio‐economic shocks.

## FUNDING INFORMATION

We acknowledge funding from the Commonwealth Secretariat for this research.

## Data Availability

Data sharing is not applicable to this article as no new data was created or analysed in this study.

## ENDNOTES

^1^
Other papers used remoteness (e.g. Márquez‐Ramos, et al., 2007), but this practice has been recently criticized (Yotov et al., 2016).

^2^
We thank the associate editor and an anonymous referee for pointing this out.

^3^
Using the income group classification from the World Bank in 2019.

^4^
We also estimated model (4) including neighboring countries, but in this case, those are mostly not statistically significant (See Table A4 in the Appendix).

^5^
Results are available on request.

## APPENDIX A

**TABLE A1:** List of Commonwealth countries included in the analysis

Partner countries	Reporter countries
Antigua and Barbuda	Antigua and Barbuda
Australia	Australia
Bahamas	Barbados
Bangladesh	Belize
Barbados	Botswana
Belize	Canada
Botswana	Cyprus
Brunei Darussalam	Eswatini
Cameroon	Ghana
Canada	Guyana
Cyprus	India
Dominica	Kenya
Eswatini	Malaysia
Fiji	Malta
Ghana	Mauritius
Guyana	Namibia
India	New Zealand
Jamaica	Pakistan
Kenya	Rwanda
Kiribati	Seychelles
Lesotho	Singapore
Malawi	South Africa
Malaysia	Uganda
Malta	United Kingdom
Mauritius	Zambia
Mozambique	
Namibia	
Nauru	
New Zealand	
Nigeria	
Pakistan	
Papua New Guinea	
Rwanda	
Samoa	
Seychelles	
Sierra Leone	
Singapore	
South Africa	
Sri Lanka	
Tanzania, United Republic of	
Tonga	
Trinidad and Tobago	
Tuvalu	
Uganda	
United Kingdom	
Vanuatu	
Zambia	

*Source*: UN Comtrade.

**TABLE A2:** Linear models estimated with OLS for the baseline specification

	(1)	(2)	(3)	(4)	(5)	(6)
Dependent variable:	lnimports	lnimports	lnimports	lnimports	lnimports	lnimports
Explanatory variables:						
lncovid measure i, t‐1	−0.039***	−0.053***	−0.016	0.002	0.000	0.004
	(0.007)	(0.007)	(0.023)	(0.003)	(0.003)	(0.009)
lncovid measure j, t‐1	0.004	0.001	−0.031	−0.012***	−0.016***	−0.023***
	(0.007)	(0.007)	(0.020)	(0.003)	(0.003)	(0.007)
lnyi	1.334***	1.333***	1.325***			
	(0.015)	(0.015)	(0.015)			
lnyj	0.869***	0.869***	0.870***			
	(0.017)	(0.017)	(0.017)			
lnpopi	−0.126***	−0.124***	−0.129***			
	(0.019)	(0.019)	(0.019)			
lnpopj	0.143***	0.143***	0.145***			
	(0.021)	(0.021)	(0.021)			
lndij	−1.020***	−1.019***	−1.017***			
	(0.025)	(0.025)	(0.025)			
Regional trade agreement dummy	0.883***	0.886***	0.882***			
	(0.046)	(0.046)	(0.046)			
Former colony dummy	0.897***	0.900***	0.891***			
	(0.124)	(0.124)	(0.124)			
Contiguity dummy	0.798***	0.800***	0.798***			
	(0.141)	(0.141)	(0.141)			
Common language dummy	0.661***	0.663***	0.664***			
	(0.062)	(0.062)	(0.062)			
lnCPIi	−0.465***	−0.460***	−0.458***	0.100*	0.099*	0.096*
	(0.097)	(0.096)	(0.096)	(0.053)	(0.052)	(0.053)
lnCPIj	−0.881***	−0.883***	−0.884***	0.151	0.154	0.148
	(0.099)	(0.099)	(0.099)	(0.096)	(0.096)	(0.096)
Observations	165,961	165,961	165,961	174,341	174,341	174,341
R‐squared	0.642	0.642	0.642	0.910	0.910	0.910
Year‐Month FE	Yes		Yes			
i and j FE	Yes	Yes	Yes			
r2	0.642	0.642	0.642	0.910	0.910	0.910
Year‐Month FE	Yes	Yes	Yes	Yes	Yes	Yes
ij FE				Yes	Yes	Yes

*Note*: Robust standard errors in parentheses. ****p* < .01; ***p* < .05; **p* < .1.

**TABLE A3:** Linear models estimated with OLS and exponential mean models with PPML

	(1)	(2)	(3)	(4)
Dep. variable	lnxij	lnxij	Trade value	Trade value
Method	OLS‐YFE	OLS_YMFE	PPML_YFE	PPMP_YMFE
lncovcai	−0.027***	−0.033***	−0.059***	−0.070***
	(0.005)	(0.006)	(0.010)	(0.013)
lncovcaj	0.016***	0.006	0.048***	0.025
	(0.004)	(0.006)	(0.012)	(0.022)
lnyi	1.350***	1.352***	0.791***	0.793***
	(0.014)	(0.014)	(0.008)	(0.008)
lnyj	0.916***	0.917***	0.591***	0.598***
	(0.015)	(0.015)	(0.025)	(0.025)
lnpopi	−0.161***	−0.160***	‐	‐
	(0.017)	(0.017)		
lnpopj	0.063***	0.066***	‐	‐
	(0.018)	(0.018)		
lndij	−1.000***	−1.002***	−0.409***	−0.409***
	(0.023)	(0.023)	(0.016)	(0.016)
Regional trade agreement dummy	0.878***	0.879***	0.100***	0.100***
	(0.043)	(0.043)	(0.021)	(0.021)
Former colony dummy	0.592***	0.594***	−0.055**	−0.053**
	(0.104)	(0.104)	(0.026)	(0.026)
Contiguity dummy	0.951***	0.947***	0.961***	0.961***
	(0.123)	(0.123)	(0.056)	(0.056)
Common language dummy	0.628***	0.626***	0.157***	0.157***
	(0.060)	(0.060)	(0.039)	(0.039)
Period = 201,902		−0.113***		−0.085
		(0.019)		(0.067)
Period = 201,903		−0.054***		0.012
		(0.020)		(0.068)
Period = 201,904		−0.017		−0.007
		(0.021)		(0.069)
Period = 201,905		0.033		0.031
		(0.021)		(0.070)
Period = 201,906		−0.092***		−0.044
		(0.021)		(0.072)
Period = 201,907		−0.010		0.027
		(0.021)		(0.071)
Period = 201,908		−0.079***		−0.048
		(0.021)		(0.074)
Period = 201,909		−0.102***		−0.022
		(0.021)		(0.072)
Period = 201,910		−0.002		0.043
		(0.021)		(0.070)
Period = 201,911		−0.092***		−0.025
		(0.021)		(0.071)
Period = 201,912		−0.113***		−0.052
		(0.022)		(0.071)
Period = 202,001		−0.088***		0.067
		(0.024)		(0.074)
Period = 202,002		−0.167***		0.053
		(0.028)		(0.094)
Period = 202,003		0.028		0.346
		(0.069)		(0.256)
Period = 202,004		−0.129		0.177
		(0.080)		(0.292)
Period = 202,005		−0.090		0.171
		(0.076)		(0.276)
Period = 202,006		−0.048		0.301
		(0.078)		(0.266)
Period = 202,007		0.011		0.412
		(0.081)		(0.278)
Period = 202,008		0.025		0.414
		(0.085)		(0.295)
Period = 202,009		0.146		0.552*
		(0.089)		(0.316)
Period = 202,010		0.191**		0.678*
		(0.097)		(0.353)
Period = 202,011		0.473***		0.241
		(0.152)		(0.325)
Year = 2020	−0.077***		0.031	
	(0.020)		(0.044)	
Observations	194,812	194,812	206,410	206,410
R2‐Overall/Ps‐R2	0.649	0.650	0.710	0.711
YFE = Year FE	Yes		Yes	
YMFE = Year‐Month FE		Yes		Yes

*Note*: Robust standard errors in parentheses. ****p* < .01; ***p* < .05; **p* < .1.

**TABLE A4:** Gravity model estimations: heterogeneous effects by income level and neighboring effects

Dependent variable:	(1)	(2)	(3)
Export value	COVID cases	COVID deaths	Stringency index
Explanatory variables:			
lncovid measure i, t‐1	−0.014***	−0.012**	−0.005
	(0.005)	(0.005)	(0.007)
lncovid measure j, t‐1	−0.012***	−0.017***	−0.035***
	(0.004)	(0.004)	(0.006)
HI*lncovid measure i, t‐1	0.002	0.003	0.002
	(0.002)	(0.003)	(0.006)
HI*lncovid measure j, t‐1	0.014***	0.020***	0.037***
	(0.002)	(0.003)	(0.005)
laglnneigcovit‐1	0.000	0.005	−0.072
	(0.012)	(0.016)	(0.050)
laglnneigcovjt‐1	−0.039	−0.051*	0.016
	(0.031)	(0.030)	(0.049)
lnCPIi	−0.116	−0.086	−0.150
	(0.112)	(0.114)	(0.122)
lnCPIj	−0.096	−0.136	−0.095
	(0.127)	(0.134)	(0.127)
Observations	145,331	145,331	145,331
Month FE	Yes	Yes	Yes
ij FE	Yes	Yes	Yes
r2_p	0.991	0.991	0.991

*Note*: Robust standard errors in parentheses. ∗∗∗*p* < .01; ∗∗*p* < .05; ∗*p* < .1.
